# Prolonged Recovery From General Anesthesia Possibly Related to Persistent Hypoxemia in a Draft Horse

**DOI:** 10.3389/fvets.2018.00235

**Published:** 2018-10-01

**Authors:** Julien Dupont, Didier Serteyn, Charlotte Sandersen

**Affiliations:** Department of Clinical Sciences, Anesthesiology and Equine Surgery, Faculty of Veterinary Medicine, University of Liege, Liège, Belgium

**Keywords:** horse, anesthesia, recovery, hypoxemia, estimated shunt fraction, hypothermia, residual drug effects

## Abstract

Horses are susceptible to developing large areas of pulmonary atelectasis during recumbency and anesthesia. The subsequent pulmonary shunt is responsible for significant impairment of oxygenation. Since ventilation perfusion mismatch persists into the post-operative period, hypoxemia remains an important concern in the recovery stall. This case report describes the diagnosis and supportive therapy of persistent hypoxemia in a 914 kg draft horse after isoflurane anesthesia. It highlights how challenging it can be to deal with hypoxemia after disconnection from the anesthesia machine and how life-threatening it can become if refractory to treatment. Furthermore, it stresses the point on the interactions between hypoxemia and other factors, such as residual drug effects and hypothermia, that should also be considered in the case of delayed recovery from general anesthesia.

## Introduction

A 9-year-old Boulonnais gelding weighing 914 kg was referred to the Equine Clinic of the University of Liege for transpalpebral enucleation of the left eye under general anesthesia. Preoperative laboratory values including total protein, packed cell volume (PCV), total hemoglobin (tHb), total and differential white blood cell count, serum creatinine, total and conjugated bilirubin, and gamma glutamyl transpeptidase were within normal limits. Physical examination was unnoticeable unless a mild tachypnoea (24 breaths/min), the patient was graded II according to the American Society of Anesthesiologists physical status.

Food, but not water, was withheld for 10 h prior to surgery. Vitamin E acetate and sodium selenite pentahydrate (VMD, Belgium; 6500 mg and 130 mg respectively), were administered IM twice, the day before and the morning of the surgery. A 12-gauge intravenous catheter was placed in the left jugular vein. Procaine penicillin (Kela, Belgium; 19.2 M UI IM), gentamicin (Franklin Pharmaceuticals, Ireland; 6 g IV), and acepromazine (Kela, Belgium; 90 mg IM) were administered 120 min before induction. Flunixin meglumine (Ecuphar, Belgium; 950 mg IV) immediately followed by xylazine (Prodivet Pharmaceuticals, Belgium; 480 mg IV) were administered as preanaesthetic medication. Anesthesia was induced with midazolam (Mylan, Belgium; 55 mg IV) and ketamine (Ecuphar, Belgium; 2 g IV) and the trachea was intubated with a 30 mm cuffed endotracheal tube (ETT) as soon as the patient was recumbent. The horse was positioned in right lateral recumbency on a padded surface and conducted to the operating room where he was connected to a rebreathing circuit.

A 20-gauge catheter was placed in the left dorsal metatarsal artery for continuous direct arterial pressure measurement and repeated arterial blood sampling. Invasive arterial blood pressure, pulse oximetry, electrocardiogram, inspired and expired percentages of oxygen and isoflurane, inspired and expired carbon dioxide partial pressures, airway pressure, and flow-volume loops were continuously recorded using a multiparameter monitor (Tafonius, Vetronics, UK). Arterial partial pressure of oxygen (PaO_2_) and carbon dioxide (PaCO_2_), pH, PCV, plasma electrolytes, arterial saturation of hemoglobin (SaO_2_), tHb, oxyhemoglobin (O_2_Hb), carboxyhemoglobin, and methemoglobin were measured with co-oxymetry (Cobas b 123, Roche, Belgium).

Isoflurane (Zoetis, Belgium) was delivered in 100% oxygen and its end-tidal percentage was maintained between 0.9 and 1.3%. Intermittent positive pressure ventilation (IPPV) was provided during the whole procedure (Tafonius) to maintain end-tidal carbon dioxide partial pressure between 35 and 45 mmHg (respiratory rate: 6–8 breaths/min, tidal volume: 9.5–10 L, I:E ratio = 1:2 and maximum peak inspiratory pressure: 35 cmH_2_O). Retrobulbar nerve block was performed with 100 mg lidocaine (AstraZeneca, Belgium) and 100 mg mepivacaine (AstraZeneca, Belgium) and auriculopalpebral nerve block with 20 mg lidocaine. Ketamine was administered three times as IV bolus (300 mg) to increase the anesthetic depth and morphine (Sterop, Belgium; 90 mg) was injected IV to control pain 82 min after induction. Partial intravenous anesthesia using ketamine (1 mg/kg/h) and midazolam (0.02 mg/kg/h) was added 25 min after induction, lasted for 95 min to be finished 80 min before the end of anesthesia. Lactated Ringer's solution (Dechra, UK) was infused during anesthesia at a rate of 5.6 ml/kg/h. Standard transpalpebral enucleation of the left eye was completed within 150 min and the total anesthesia time was 200 min.

Heart rate was comprised between 35 and 45 beats/min. Systolic, mean and diastolic arterial blood pressures ranged between 85 and 120, 65 and 95, and 50 and 85 mmHg, respectively. The first arterial blood gas revealed mild hypoxemia (PaO_2_ 64 mmHg). Salbutamol (Sandoz, Belgium; 1.9 mg) was administered through the ETT with a metered-dose inhaler 37 min after induction. However, the second arterial blood sample showed only a small improvement (PaO_2_ 73 mmHg). Therefore, an alveolar recruitment maneuver (ARM) was performed 47 min after induction. Practically, it consisted in interrupting IPPV during inspiratory phase and applying a continuous positive airway pressure (CPAP) of 50 cmH_2_O during 45 s. Afterwards, IPPV was resumed and a positive end-expiratory pressure (PEEP) of 10 cmH_2_O was maintained until the end of the procedure. Ulterior arterial blood samples showed a progressive improvement and hypoxemia was solved (PaO_2_ 80, 103, 153, and 203 mmHg at 3, 34, 61, and 90 min post-recruitment maneuver, respectively). Data from blood gas analysis are displayed in Table 1.

**Table 1:** Peripheral arterial and venous blood samples analyzed during anesthesia (A), when recumbent during recovery (R) and after standing (S).

**Time**	**A/R/S**	**Patm**	**FiO_2_**	**pH**	**Hba**	**PaO_2_**	**SaO_2_**	**PaCO_2_**	**PvO_2_**	**SvO_2_**	**PvCO_2_**	**Lact**	**CK**	**PAO_2_**	**F-shunt_e_**
31 min	A	742.6	0.66	7.396	10.5	64.3	94.2	49.3	/	/	/	/	/	397.5	35
42 min (after salbutamol)	A	742.7	0.71	7.380	9.3	73.0	96.0	44.9	/	/	/	/	/	437.8	32
50 min (after ARM)	A	742.8	0.80	7.379	9.9	79.7	96.6	49.8	/	/	/	/	/	494.4	33
1 h 21 min	A	742.7	0.82	7.361	10.5	103.3	98.0	51.0	/	/	/	/	/	506.7	31
1 h 48 min	A	742.7	0.84	7.364	9.8	153.1	98.8	52.0	/	/	/	/	/	519.4	27
2 h 17 min	A	742.8	0.85	7.384	9.7	203.4	98.9	51.7	/	/	/	/	/	526.8	25
5 h 42 min	R	742.0	0.30	7.501	/	40.5	/	36.4	/	/	/	/	/	163.0	/
5 h 46 min	R	741.8	0.30	7.441	/	/	/	/	20.7	40.2	47.7	4.2	787	/	/
10 h 38 min	R	741.8	0.30	7.401	13.2	48.1	84.9	46.0	/	/	/	3.2	/	150.9	46
13 h 27 min	R	742.0	0.30	7.377	12.8	57.2	91.0	44.1	/	/	/	/	/	153.4	35
21 h 36 min	R	740.7	0.21	7.340	/	/	/	/	25.5	43.0	51.4	2.8	/	/	/
23 h 15 min	R	740.9	0.21	7.386	13.4	46.7	85.8	39.8	/	/	/	/	/	96.0	44
27 h 27 min	R	740.8	0.30	7.401	/	61.6	/	34.1	/	/	/	/	13,000	165.5	/
34 h 44 min	R (after S)	741.8	0.21	7.424	11.3	49.0	89.0	41.2	/	/	/	3.8	/	94.4	34
45 h 59 min	S	743.4	0.21	7.454	11.8	38.1	78.6	41.2	/	/	/	0.9	3,788	94.7	51

*Patm, atmospheric pressure (mmHg); FiO_2_, inspired oxygen fraction (mmHg); Hba, arterial hemoglobin concentration (g/dl); PaO_2_, arterial oxygen partial pressure (mmHg); SaO_2_, arterial hemoglobin oxygen saturation (%); PaCO_2_, arterial carbon dioxide partial pressure (mmHg); PvO_2_, venous oxygen partial pressure (mmHg); SvO_2_, venous hemoglobin oxygen saturation (%); PvCO_2_, venous carbon dioxide partial pressure (mmHg); Lact, lactates (mmol/L); CK, creatine kinase (UI/L); PAO_2_, alveolar oxygen partial pressure (mmHg); F-shunt_e_, estimated shunt fraction (%); ARM, alveolar recruitment maneuver*.

$$\begin{array}{l} PAO_{2}=FiO_{2}*(PAtm-PH_{2}O)]-(PaCO_{2}/0.8).\\ F-shunt_{\text{e}}=\left\{\frac{\left[\left(1,36*Hba*\left(1-SaO_{2}\right)\right)+\left(0,0031*\left(PAO_{2}-PaO_{2}\right)\right)\right]}{\left[1,36*Hba*\left(1-SaO_{2}\right))+\left(0,0031*\left(PAO_{2}-PaO_{2}\right)\right)+3,5\right]}\right\}*100 \end{array}$$

**Figure 1:**
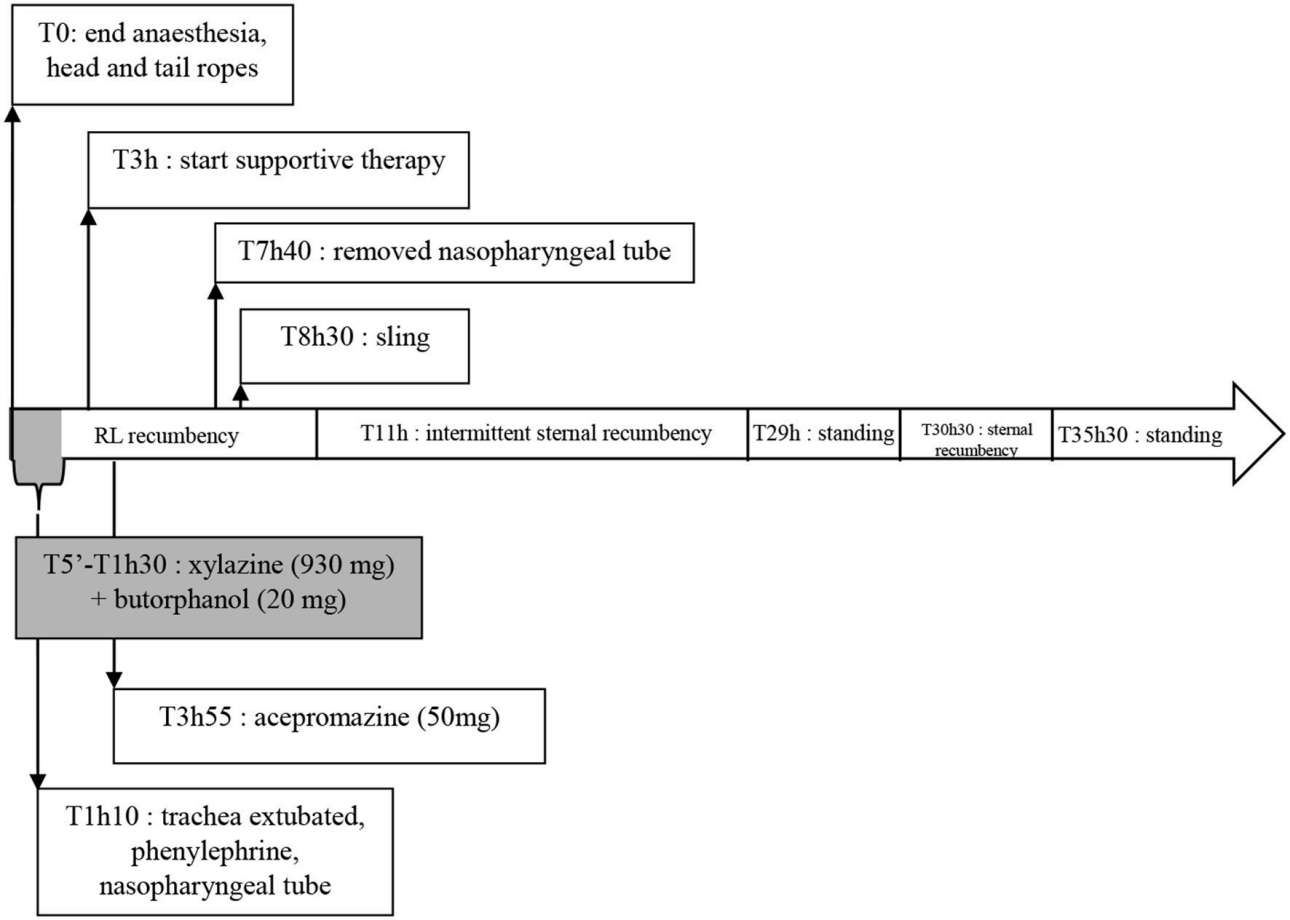
Time line of the main events that happened during recovery. RL, right lateral.

At the end of surgery, the horse was placed in right lateral recumbency in a rubber floored and heavily padded recovery stall. The ETT was secured and oxygen (15 L/min) was administered through it. The horse became extremely agitated 5 min after isoflurane discontinuation and needed to be sedated nine times with xylazine (total dose: 930 mg IV) and butorphanol (Ecuphar, Austria; 20 mg IV) to avoid self-inflicted traumas. The trachea was extubated 70 min after the end of anesthesia. Because of snoring, 10 ml of a solution of 0.5% phenylephrine (Bausch and Lomb, Belgium) was instilled in each nostril, a 16 mm nasopharyngeal tube was inserted through the left nostril and oxygen therapy was continued. He removed the nasopharyngeal tube during one of his violent uncoordinated movements but, because he was not snoring anymore, the oxygen hose (15 L/min) was placed directly in the nose to the pharynx and maintained in place whenever it was possible. Following the nine unsuccessful attempts to sedate the horse with xylazine, and as he was still not standing at that time, acepromazine was administered (50 mg IV), providing light but longer sedation (Figure 1).

Supportive therapy was started in the recovery stall as the recovery period exceeded 180 min. It consisted in fluid therapy with lactated Ringer's solution (11 ml/kg/h) supplemented with calcium gluconate (Dechra, Belgium), magnesium sulfate (Sterop, Belgium), and potassium chloride (Braun, Germany). Alternatively, sodium chloride 0.9% (Aguettant, Belgium) was infused according to plasma electrolytes measurement (sodium 137.2 mmol/l; chloride 98.6 mmol/l). Furthermore, nutrition support was provided with glucose 50% (Baxter, Belgium; 2mg/kg/min). Inotropic support with dobutamine (Mylan, Belgium; 1 μg/kg/min) was also added as the pulse was weak. Analgesia was provided with morphine (90 mg IM) TID and flunixin meglumine (950 mg IV) BID. The horse was also given a single dose of dexamethasone (Eurovet, Holland; 92 mg IV) 285 min after isoflurane discontinuation. Regular physical examinations were performed: the pulse became stronger after fluid therapy and inotropic support were initiated. Mucosa were pink and refill capillary time was prolonged up to 4 s. He presented tachycardia (50–64 beats/min) with arrhythmias and mild tachypnoea (22–28 breaths/min). Hypothermia, up to 33.4°C, was efficiently treated by covering him with blankets. Furthermore, he showed nystagmus and an obtunded pupillary light reflex. As the recovery period abnormally prolonged, concern was raised about generalized post-anaesthetic myopathy. However, the soft and non-painful muscle palpation, the normal gross appearance of urine and serum creatine kinase levels (Table 1) did not advocate for this condition.

A sling was put on the horse 8.5 h after entering the recovery stall, in addition to head and tail ropes, which were attached from the beginning, to lift him up with the hoist. Unfortunately, we did not manage to get him to standing position after having tried several times. Because he was very agitated and heavy, we also failed to change recumbency and he spent 14.5 h (including surgery) in right lateral recumbency. At 11 h after anesthesia, he managed to get and stay intermittently in sternal recumbency. Twenty-nine hours after the end of anesthesia, the horse finally stood up without any assistance. The right front leg was painful and he could not bear any weight on it, he kept standing for 90 min before lying down again. The horse finally stood up 5 h later and he was moved out of the recovery stall. The likely radial neuropathy and/or triceps myopathy were treated by administering acepromazine (46 mg IM) TID, morphine (90 mg IM) TID, ketamine (460 mg IM) QID and flunixin meglumine (950 mg IV) BID. Low molecular weight heparin (Sanofi, Belgium; 150 mg) was administered SC SID and supportive hoof bandages were placed on both front legs to prevent laminitis. Moreover, the horse presented paralysis and distortion of the nose and lips to the right side, most likely due to paralysis of the buccal branch of the right facial nerve, which spontaneously returned to normal upon discharge. Furthermore, the horse developed a surgical wound dehiscence due to infection, which successfully healed after a second surgery under standing sedation.

## Background

### Perioperative respiratory complications

Horses are susceptible to quickly developing large areas of atelectasis and a consequent large pulmonary shunt causing significant impairment of gas exchange during recumbency and anesthesia (1–4). Pulmonary shunt has been estimated at 1% in standing horses, extending to 19%, and 33% in anesthetized laterally and dorsally recumbent horses, respectively (5, 6).

Compression atelectasis is the major type of atelectasis in anesthetized horses (6). Recumbency changes distribution of ventilation by reducing lung volume and altering the pleural pressure gradient to such an extent that the peripheral airways close in the dependent regions of the lung where closing volume exceeds functional residual capacity (2, 5). Body weight and body shape both influence PaO_2_ and alveolar arterial oxygen gradient (Aa gradient). Indeed, light-weight animals which are tall with large thoracic circumference and flat belly better maintain oxygenation (7–9).

Moreover, characteristics of the pulmonary artery in the caudodorsal regions of the lung alter the hypoxic vasoconstrictive response to alveolar hypoxia, leading to preferential caudodorsal lung perfusion and larger ventilation perfusion mismatch regardless of the posture (3, 10, 11). In addition, most anesthetics, and particularly inhalants, deeply reduce or even abolish hypoxic vasoconstriction and flow redistribution (12).

Consequently, there can be prolonged periods of hypoxemia during general anesthesia in horses (4, 13–16).

Since ventilation perfusion (V/Q) mismatch persists into the postoperative period (17), hypoxemia remains an important concern in the early recovery period, especially when breathing room air reduces the inspired oxygen fraction (FiO2), requiring vigilant monitoring and oxygen supplementation (18). Nasotracheal insufflation of oxygen at a flow rate of at least 15 L/min immediately after disconnection from the anesthesia machine efficiently improves PaO_2_ and relieves hypoxemia in the recovering horse (18). Furthermore, horses auto-recruit their lungs by inspiratory breath holding following recovery from general anesthesia, possibly reflecting a compensatory mechanism to counteract persistent atelectasis (19).

### Residual drug effects

Ketamine infusion is commonly used to balance inhalational anesthesia and midazolam infusion is frequently added to reduce the central excitatory effects of ketamine (20). Nevertheless, common concerns are often raised about their negative influence on recovery quality after prolonged infusion and the difficulty to predict the pharmacokinetics and pharmacodynamics of drugs combinations.

The pharmacokinetics of midazolam has only been described in conscious horses (21). Redistribution is responsible for the termination of its clinical effect and accumulation in peripheral compartment is highly probable.

Two studies described the pharmacokinetics of racemic ketamine after infusion in conscious horses (22, 23). They both mentioned that the pharmacokinetics of ketamine after infusion was different from those described after single bolus. Moreover, both studies stressed the point that premedication or concurrent administration of inhalants or other anesthetics were known to influence the pharmacokinetics of ketamine. Ketamine undergoes rapid metabolism to norketamine, whose redistribution and metabolism are slower than for parent drug. Nevertheless, the contribution of ketamine's metabolite to its pharmacological effects is unknown in the horse.

Intraoperative administration of ketamine has been shown to be a significant predictor of faster recovery time, indicating that these horses were kept at a lighter plane of anesthesia, and consequently had less isoflurane accumulation (24).

### Inadvertent perioperative hypothermia

Several studies reported that hypothermia prolong time to standing in horses (24–26).

Drug metabolism relies on enzymatic reactions that may be altered by hypothermia. Hypothermia can therefore prolong recovery time (27).

## Discussion

### Perioperative respiratory complications

#### Assessment of oxygenation

##### diagnostic tools

Usually, clinical assessment is sufficient to monitor most recoveries. However, different indices have been described to assess oxygenation. Venous admixture (Qs/Qt) is the most accurate but requires mixed venous blood collected from the pulmonary artery. Estimated shunt fraction (F-shunt_e_) is a content-based index that is calculated from peripheral arterial blood and has the best agreement with Qs/Qt (28).

##### perioperative hypoxemia and atelectasis

Arterial blood gas analysis revealed mild hypoxemia at the beginning of the anesthesia period (PaO_2_ 64 mmHg). Inhalation of salbutamol and ARM followed by PEEP reverted hypoxemia (PaO_2_ up to 203 mmHg at the end of the anesthesia period). During anesthesia, F-shunt_e_ lay between 25 and 35%. In the recovery stall, PaO_2_ was between 41 and 62 mmHg, corresponding to mild to moderate hypoxemia, and F-shunt_e_ lay between 34 and 46% (Table 1). These calculated values of shunt percentage were largely superior to the expected value of 19% in anesthetized laterally recumbent horses (5, 6), suggesting a large pulmonary shunt responsible for hypoxemia in that horse. Moreover, PaO_2_ 10 h after standing was 38 mmHg, corresponding to severe hypoxemia and F-shunt_e_ was 51%.

#### Prevention and treatment of hypoxemia

##### oxygen supplementation

These measurements showed that, despite oxygen insufflation immediately after disconnection from the anesthesia machine, hypoxemia developed during recovery. Administering oxygen in ETT creates a combination of room air and oxygen leading to FiO_2_ between 30 and 70% (29). The further distal in the airway oxygen is delivered, the greater the increase in FiO_2_ for a particular oxygen flow rate (29). However, the horse could not bear the nasopharyngeal tube and was sometimes so agitated that the anesthetist did not manage to maintain the oxygen hose in his nose continuously, preventing from proper oxygen insufflation. Furthermore, the degree of improvement in PaO_2_ depends on the increment in FiO_2_ and on the degree of V/Q mismatch (29). Nevertheless, increasing FiO_2_ during anesthesia is generally unsuccessful in correcting hypoxemia since much of the impairment in gas exchange results directly from shunt (30). Unfortunately, we did not measure the real FiO_2_ during recovery and considered 30% for F-shunt_e_ calculation, which might be a potential source of imprecision.

##### lung recruitment

Open lung concept consists in, first, applying a high PIP to reinflate atelectatic lungs, which is also referred as ARM, and, second, maintaining a PEEP to prevent re-collapse. Indeed, once a critical amount of atelectasis is present in the equine lung, it is difficult to recruit that portion of the lung using traditional ventilation strategies (29) and PIP of up to 80 cmH_2_O and PEEP of up to 30 cmH_2_O are required (31–36). Two strategies are commonly used: either a stepwise incremental and decremental PIP and PEEP titration; or a sustained high-pressure maneuver followed by a predetermined PEEP. Although high airway pressures inevitably induce cardiovascular and pulmonary side effects, the first technique may present two main advantages: first, by allowing the cardiovascular system to better adapt to higher intrathoracic pressures; and second, by using the lowest PEEP required to keep recruited alveoli open (32, 34, 35, 37, 38). Optimal PEEP for each patient is best titrated by monitoring PaO_2_ or the compliance of the dependent lung assessed by electrical impedance tomography (35). Despite that the sustained high-pressure ARM followed by predetermined PEEP used in this case resulted in reduction of pulmonary shunt and resolution of hypoxemia, these improvements did not extend in the recovery period. These observations suggested re-collapse as soon as positive airway pressure is lost, which is conflicting with studies reporting applications of modified open lung concept techniques. It is technically difficult to provide PEEP after disconnection, but, in theory, it may have limited de-recruitment (32, 33, 36, 37).

##### auto-recruitment

It has been reported that horses auto-recruit their lungs by inspiratory breath holding until 5 h after standing (19). Nevertheless, this seemed not to be the case for this horse because pulmonary shunt and hypoxemia worsened after standing for 10 h. The reason why he did not manage to recruit his lungs is not clear. Obviously, the horse did not present a clinical picture compatible with a severe lung pathology such as pulmonary edema, pneumothorax, or pulmonary embolism. However, gene expression quantification has shown that mechanical ventilation, either IPPV (PIP of 20 cmH_2_O) or stepwise ARM combined with PEEP (PIP of up to 60 cmH_2_O and PEEP of 20 cmH_2_O), might be responsible for an early inflammatory state in the lungs. Indeed, these ventilation strategies both increased markers possibly associated with lung injuries without being related to any histological lesion nor any modification of total and differential cell counts in bronchoalveolar lavage fluid (39). Therefore, we cannot exclude that the ventilation strategy applied to this horse, and combining IPPV (PIP of up to 35 cmH_2_O) and sustained high-pressure ARM followed by predetermined PEEP (CPAP of 50 cmH_2_O and PEEP of 10 cmH_2_O), might not have caused alterations in the lungs that prevent from auto-recruitment.

Moreover, the higher weight of that draft horse might have played a role as for example prolonged atelectasis has been demonstrated in morbidly obese patients (40).

##### body weight

As body weight and body shape both influence PaO_2_ and Aa gradient (7–9), body weight might have played a role in the development of atelectasis, compression atelectasis being the major type of atelectasis in anesthetized horses (6).

##### duration of anesthesia

Duration of anesthesia is known to influence the incidence of episodes of hypoxemia in humans (41, 42). The total anesthesia time was 200 min, which is much more than usual for transpalpebral enucleation and might have contributed to hypoxemia.

##### anaesthetics

Anaesthetics may alter cardiorespiratory function and therefore negatively influence gas exchange. However, morphine and butorphanol have not produced clinically significant cardiorespiratory impairments, maintaining PaO_2_ (43, 44). Similarly, acepromazine has improved arterial oxygenation by reducing V/Q disturbance and fall in PaO_2_ associated with general anesthesia (45).

#### Hypoxemia-related postoperative complications

##### delayed recovery

Inhalants attenuate autoregulation of cerebral blood flow (46). Inhalants and hypoxemia can be involved in brain injury and consequent altered cognition (47), potentially explaining nystagmus and obtunded pupillary light reflex. They might therefore affect recovery quality. However, hypoxemia is aggravated by repeated attempts to stand (48). Moreover, hypoxemia reduces the strength of muscle contraction (49). In that case, reduced cardiac output, probably added to hypoxemia, led to further decrease in tissue oxygen delivery. Indeed, pulse became stronger after initiation of supportive therapy, suggesting improvement in circulatory function.

### Residual drug effects

The use of midazolam (loading dose 0.04 mg/kg and infusion rate 0.02 mg/kg/h) and ketamine (loading dose 2.5 mg/kg and infusion rate 1 mg/kg/h) in sevoflurane-anesthetized horses has been described (50). All horses recovered satisfactorily but showed mild ataxia for 15–20 min, probably due to midazolam. As doses that we used were comparable to those described in this study; and as infusion lasted for a shorter duration (95 vs. 126–190 min) and was discontinued well before switching off inhalant (80 vs. 0 min), it is less likely that neither midazolam nor ketamine prevented our horse from standing.

Some studies described ketamine infusions in halothane-anesthetized horses (51–53), using loading doses ranging from 2.2 to 2.4 mg/kg and infusion rates from 2.0 to 2.8 mg/kg/h. All recoveries were considered acceptable and comparable of those observed with halothane only. We used comparable loading dose but our infusion rate was two to almost three times less than that described in these studies. Furthermore, the maximum infusion time was shorter (95 vs. 127 min) and it was stopped much more ealier than inhalant (80 vs. 0–15 min). Consequently, it is less likely that ketamine might have interfere with recovery in our case.

Although its pharmacological activity has not been described yet in horses, norketamine accumulation is still a concern. Its implication in the nystagmus and the obtunded pupillary light reflex that we observed can not be ruled out.

### Inadvertent perioperative hypothermia

Hypothermia might be partly responsible for prolonged recovery. Indeed, it may play a part in midazolam and ketamine accumulation by reducing their metabolism. Moreover, by reducing baroreceptor function and cardiomyocytes contractility, hypothermia may be involved in the low cardiac output suspected in our case as well as in the cardiac arrhythmias that we noticed (54, 55). Furthermore, it may decrease central nervous system function and alter cerebral perfusion, potentially leading to the altered cognition that we observed (56). In addition, hypothermia and consequent shivering might have worsened hypoxemia by greatly increasing oxygen consumption. In our case, body temperature should have been more closely monitored during anesthesia and recovery; and hypothermia should have been aggressively treated as soon as it appeared.

## Concluding remarks

When facing complicated recovery, all the potentially contributing factors should always be contemplated. In this case, hypoxemia, residual drug effects, and hypothermia are three relevant factors that might have prolonged the period of time elapsed before the horse stood up. Indeed, although hypoxemia is a common complication in equine anesthesia and should be considered, residual drug effects and hypothermia should not be misregarded as they might have been responsible for rough recovery even without hypoxemia.

## Ethics statement

While signing the hospitalization contract, the owner is aware and accepts that data concerning his animal might be used for scientific purposes without further consent, guaranteeing confidentiality and anonimity of the owner.

## Author contributions

JD, DS, and CS were involved in clinical management of the case including data treatment and interpretation. All authors were involved in the preparation of the manuscript.

### Conflict of interest statement

The authors declare that the research was conducted in the absence of any commercial or financial relationships that could be construed as a potential conflict of interest.
